# Supplementary material for: Treatment of refractory poly articular course juvenile idiopathic arthritis with tofacitinib: Extended experience from Bangladesh
Source: PLoS One. 2025 Jan 24;20(1):e0312174. doi: 10.1371/journal.pone.0312174 (PMC11759992; doi:10.1371/journal.pone.0312174)
Supplement: Attachment — Submitted filename: Response to reviewers.docx [file pone.0312174.s002.docx]

**Response to reviewers**

30.08.2024

Dear Academic Editor,

We thank the reviewers for their generous comments on the manuscript and have edited the manuscript to address their concerns.

We have corrected sentence structure, punctuation and tried to put appropriate word. The areas we have changed marked with yellow colour.

We believe that the manuscript is now suitable for publication in your journal.

Regards,

Dr. Kazi Iman

kaziiman28@gmail.com
